# The Clinical, Pathological and Radiological Aspects of Infection of the Teeth and Gums

**Published:** 1923-04

**Authors:** William Willcox

**Affiliations:** Physician to St. Mary’s Hospital


### THE CLINICAL, PATHOLOGICAL AND RADIOLOGICAL ASPECTS OF INFECTION OF THE TEETH AND GUMS

BY SIR WILLIAM WILLCOX, K. C. I. E., C. B., C. M. G., M. D., F. R. C. P.
Physician to St. Mary's Hospital

### HISTORICAL

Septic absorption from the teeth and gums must date from the earliest period of man's history, for the examination of ancient skulls shows that dental sepsis has ever been present. The clinical symptoms resulting from infection of the teeth and gums must have afflicted the human race during its whole existence, and it is very remarkable that the causation of these symptoms escaped recognition for so long a period. The recognition of the clinical symptoms resulting from oral sepsis is quite modern.

Disease of the teeth and gums was described by H. A. Fauchard in 1740, and from that date much has been written on the subject. In 1875, John T. Riggs gave a full description of the disease in an address read before the American Academy of Dental Surgery on "Suppurative inflammation of the gums and absorption of the gums and alveolar process," and the disease "pyorrhea alveolaris" is often called "Rigg's disease."

The next advance in knowledge resulted from the development of bacteriology and its application to the investigating of the varieties of organisms causing dental sepsis. Galippe and Professor Miller did valuable pioneer work in this respect from 1884 to 1894. The work of Pasteur and Lord Lister on the great influence of bacterial infection in the causation of disease prepared the way for the recognition of the far-reaching effects of the toxic absorption resulting from the organisms found to be associated with dental infections.

Dr. William Hunter, a distinguished Fellow of this Society, was the pioneer amongst physicians in recognizing "the relation of dental diseases to general diseases," and he read an important paper bearing this title before the Odontological Society of Great Britain in 1900. It is not too much to say that the appreciation today of dental sepsis as one of the commonest and most important causes of general diseases is very largely due to the unceasing and untiring work of Hunter in this department of medicine.

THE BACTERIA CAUSING INFECTION OF THE TEETH AND GUMS

Professor Miller, of Berlin, in 1900, carried out a series of brilliant researches on the bacteria found in connection with dental infections. He found no less than twenty different bacteria in twelve cases of pyorrhea; amongst them were streptococci, staphylococci, bacilli of various kinds, and deptothrix. Recently, J. McIntosh, Warwick James and P. Lazarus-Barlow have isolated a bacillus which they believe to be the cause of dental caries and have named bacillus acidophilus odontolyticus.

In this discussion we are mainly concerned with the organisms occurring in dental infections, the absorption of which, or their toxins, gives rise to general disease. There seems to be no doubt that it is the streptococcus infections which are almost entirely responsible. The streptococci found in mouth infections are usually classified into three groups, from their behavior when grown on media containing blood.

1. The Hæmolytic group cause severe toxæmia, and are found in the anæmias resulting from dental sepsis.

2. The Viridans group includes those streptococci which are associated with rheumatic affections. Streptococcus salivarius and streptococcus fæcalis belong to this group. They are both toxic, producing general toxæmic symptoms, and may give rise to arthritis, fibrositis, and other rheumatic affections. Arthritis and cardiac lesions have followed the inoculation of rabbits with the living organism in either case. Each of them has been found in malignant endocarditis.

3. The indifferent group of Streptococci are not toxic to guinea-pigs, and their association with rheumatic conditions is doubtful.

4. Gram-negative cocci are found associated with dental infections, but they are not usually toxic, and some of these types have probably been described as staphylococci in earlier writings.

5. Staphylococci are not usually found in dental infections, but often in the postnasal space.

In the infections of the teeth and gums the same streptococcus is not necessarily found in different cases, nor can the local disease be constantly transmitted from an infected patient by inoculation of the healthy gums of another person; so that Koch's postulates of a specific infection are not satisfied. The infecting organisms are variable and the infection may be mixed, so that the problem of dental sepsis is a complicated one.

### EVIDENCE FOR THE CONCLUSION THAT THE TEETH AND GUMS ARE THE SOURCE OF INFECTION.

It must not be hastily assumed because a patient is suffering from symptoms such as arthritis, fibrositis, anæmia, or some affection which is commonly associated with dental sepsis, that the teeth are the cause of the illness. Every case must be approached with an open mind and no preformed opinion.

There are many other foci of infection which may produce diseases identical with those resulting from dental sepsis; in other words, a streptococcal toxæmia may be due to a great many causes other than dental. Thus the tonsils, the naso-pharynx, the intestine, and the urogenital tract may all be foci of infection for pathogenic streptococci or other organisms. Careful examination should always be made to determine if these other foci of infection may safely be excluded.

The practice of extraction of apparently healthy teeth merely because a patient is suffering from a disease which is often due to a dental infection can not be too strongly condemned. There should always be strong evidence that the teeth are primarily and directly responsible for the disease in other organs before their extraction is justifiable.

### THE SIGNS OF UNHEALTHY CONDITIONS OF THE TEETH AND GUMS

The presence of gingivitis is shown by an unhealthy spongy condition of the gums, which bleed readily and have a congested free margin. Often congested hyperplastic projections occur between the teeth. These are particularly well marked in scorbutic conditions and have been described as "buds." Tartar and dirty collections may be present between the free surface of the gums and teeth. Pressure on the gums may cause pus to ooze up from between the gums and teeth if pyorrhœa is present. Local swellings of the gums due to periosteal abscess may occur. The teeth themselves may show marked caries with gingivitis around them. An important clinical symptom often present is a sweet sickly smell of the breath.

In a large number of patients who habitually clean their teeth carefully little or no evidence of a dental infection can be found from an external examination; this point can not be too strongly emphasized. On many occasions I have seen teeth and gums, apparently perfectly healthy, in which radiographic examination disclosed dangerous peri-apical necrotic lesions. Sir Frank Colyer truly says, in his book on "Chronic General Periodontitis":

"It is not safe to judge the extent of the disease from clinical appearances only, and it is necessary to call in the aid of skiagraphy in order to ascertain how far bone destruction has proceeded."

The gums may appear healthy, and yet there may be an extensive disease of the alveolar process around the roots of the teeth, and considerable bone destruction, the involved area being heavily infected with pathogenic streptococci. The grave clinical effects resulting from infected bone are well known, and from observation of a large number of cases which have been carefully investigated by bacteriological and radiological methods I am strongly of opinion that the general clinical effects produced by dental infections are accounted for by the extent and nature of the disease of the bone in the neighborhood of the teeth, rather than in the gums or teeth themselves, though these latter are the primary causes of the bone disease.

It can not be insisted upon too strongly that in every case of illness in which the teeth may be primarily responsible—even if external appearances of the teeth and gums are healthy—a radiographic examination should be made to insure that the alveolar process around the teeth is also healthy.

### RADIOGRAPHIC EVIDENCE.

Careful photographs of the teeth and surrounding jaw, taken so that two or three teeth only are included in each plate, will show in detail the exact condition of the roots of the teeth and surrounding bone.

The periodontal membrane of a tooth may be swollen, and the alveolus may show superficial erosion, such as occurs with advanced age, but the most important evidence of all is the presence of necrosis of bone in a more or less spherical area around the apex of the tooth.

These apical lesions are commonly called "apical dental abscesses." The term is a bad one because there is no pus present, and, most important of all, they give rise to no pain. The term "apical granulomata" has been used; this also is bad, because the microscopical appearances are not those of a granuloma.

When a tooth is extracted with an apical dental lesion, a mass of solid gelatinous substance is found adherent to the apex. This contains pathogenic streptococci and necrotic substance with very few leucocytes. The term "peri-apical bone necrosis" would accurately describe the condition actually present.

In my opinion the "peri-apical bone necroses"—the so-called "apical dental abscesses"—are the most serious lesions found in connection with dental sepsis, and it is these which give rise to the gravest general disease resulting therefrom. It is probable that from these lesions there is a constant flow into the blood stream of either virulent streptococci or their toxins, and the anatomical position of the lesions prevents an adequate supply of leucocytes and bactericidal body fluids to the part.

An interesting example of the deadly effect of even a small peri-apical bone lesion may be cited here.

### CASE I

In 1919, a patient, aged 46, had been under my care for some months, suffering from the effects of long residence in the tropics. He was in fairly good health and then developed an irregular pyrexia. No evidence of malaria was found and the pyrexia did not respond to quinine. A systolic murmur was present in the apical cardiac area, which was of old standing. A blood culture made by Dr. J. Matthews showed a streptococcal infection. Although the teeth had been pronounced perfectly healthy by an experienced dental surgeon an X-ray examination was made and this revealed the presence of a "peri-apical bone necrosis" round one of the lower central incisor teeth. Extraction of the affected tooth showed a marked infection with streptococci, identical in characters with those found in blood culture. The patient developed additional cardiac valvular lesions and was seen in consultation with me by Sir William Hale-White. The condition was obviously one of infective endocarditis, and this subsequently terminated fatally.

If lesions of this kind are present, the maintenance of health is obviously impossible; some serious general disease is bound to result if it has not already appeared. In my opinion if a lesion of this kind be present no compromise is permissible—the affected tooth must be extracted.

Gardner, of America, has asserted that extraction of the tooth is not a sufficient safeguard and advises a preliminary trephining of the alveolar process over the peri-apical lesion. The diseased area is then curetted, the tooth extracted and the infected area sterilized by the application of a suitable antiseptic. In this country the simple extraction of the affected tooth is, I think, considered adequate, and trephining and curettage are not thought necessary.

Where "peri-apical bone necroses" are present the affected tooth is usually dead, and this is an important indication. Attention must here be called to the very common occurrence of this dangerous lesion in connection with crowned teeth. So common is this occurrence in my experience that no hesitation in condemning the procedure of crowning teeth.

### DOES THE SIMPLE EROSION OF THE SURFACE OF ALVEOLAR PROCESS AROUND TEETH CALL FOR EXTRACTION?

Each case must be judged on its merits. If there are no obvious symptoms of streptococcal toxæmia, I should say that the answer to this question is No. The presence of gingivitis without bone disease around the teeth should be amenable to treatment and does not necessarily call for extraction.

The presence of pyorrhœa alveolaris does not of itself demand extraction of the teeth. This should only be decided upon after considering the radiographic evidence in conjunction with the local symptoms of pyorrhœa. Also the gravity of the constitutional toxic symptoms resulting from the pyorrhœa will be an important factor. It must be remembered that sometimes pyorrhœa alveolaris may be a secondary symptom of some primary disease, such as dysentery, colitis, scurvy, etc., and if the general disease is cured the cure of the pyorrhœa may follow naturally.

The general factors which influence the effects of dental sepsis are:

1. *The Virulence of the Organism.* Just as in other pathological infections—diphtheria, for example—a small lesion, if the organism is virulent, may produce very severe effects, or an extensive local dental lesion may give rise to little constitutional disturbance.

2. *The Amount of Toxins Absorbed* into the circulation is an important factor. Everyone who is familiar with hospital out-patient practice has been impressed by the appalling dental sepsis observable by the naked eye in patients with no constitutional symptoms therefrom. The reason must be that there is free discharge of the toxins produced. On the other hand, an invisible deep-seated lesion with healthy gums may be associated with the most severe constitutional effects. The "time factor" is important. If there is only slow absorption the toxic effects will be slighter than with rapid absorption. The whole question is one of dosage with toxin.

3. *The Resistance of the Patient.* This is a most important factor. Some patients, from constant absorption of toxins, become extremely sensitive, and a condition of anaphylaxis results. The recent work of Sir Almroth Wright on septicæmia is most interesting in this respect, since he has shown that in certain conditions of septicæmia the patient is capable of developing bactericidal substances. Julius A. Toren, of Chicago, showed (1921) that in certain cases of dental infection and gingivitis a leucopenia occurred, and he regarded this as an anaphylactic phenomenon and a signal of danger. He concluded that in this condition extraction of many teeth was dangerous and advised removal of not more than one at a time. This observation has a very important practical bearing.

### SECONDARY INTESTINAL INFECTION

Where dental sepsis is present and it has given rise to general disease, examination of the stool or intestinal washings shows in practically every case a marked preponderance of streptococci similar to those present in the dental lesion. This is an important factor in the consideration of the general effects produced by dental sepsis, for in most of these cases there is a secondary intestinal infection which may of itself cause similar general disease. This explains why in advanced cases of arthritis the disease may progress when the primary dental focus in infection has been eradicated.

### THE GENERAL DISEASE CAUSED BY INFECTION OF THE TEETH AND GUMS

Infection of the teeth and gums by reason of the streptococcal infection arising therefrom is undoubtedly one of the greatest sources of disease of adult life.

Acute streptococcal septicæmia and septicopyæmia have not infrequently arisen from dental sepsis. The risk of this dangerous complication must always be borne in mind in connection with the extraction of infected teeth in patients whose resistance to streptococcal infection is low. As already mentioned, a leucopenia is a danger signal.

Toxæmia is commonly associated with dental sepsis. It may be chronic, subacute, or acute, chronic toxæmia is present in a great many of these cases, and in all where some general disease has resulted from the primary dental infection. There is a feeling of malaise and general ill-health, a tendency to exhaustion on slight exertion, a pale and muddy complexion is common, and often some general pains in the hands and feet indicate an irritation of the peripheral nerves; frequently some symptoms of fibrositis or threatening arthritis occur. Insomnia, headache, and dyspeptic symptoms are common. In such cases a careful radiographic examination of the teeth should always be made.

Subacute toxæmia may give rise to intermittent pyrexia, with general constitutional symptoms of ill-health, extending over months and years.

CASE II

F., aged 32, recently seen by me had had an irregular pyrexia for three years, the temperature in the evening, commonly being 100° or 101°, and marked constitutional symptoms of ill-health were present. Extensive bacteriological investigations had been carried out, with negative result, beyond the finding of a streptococcal infection in the stools. Vaccine treatment had been of no avail. An X-ray examination of the teeth showed two extensive peri-apical necroses, although to external appearances the gums and teeth were healthy and no toothache had occurred.

Extraction of the infected teeth by Sir Frank Colyer resulted in the complete disappearance of the pyrexia and restoration of good health.

In some cases pyrexia and general symptoms of ill-health are followed by profound acute toxæmia, and a condition of stupor, delirium, and coma result.

CASE III.

M., aged 57, had had malaise and irregular pyrexia due to septic teeth. In consequence of this six teeth were extracted on July 24, 1921. The operation was followed by a condition of profound toxæmia, the patient being in a "typhoid state" with pyrexia, stupor, and low delirium; there was slight splenic enlargement and a macular rash.

All tests for enteric or typhus infections were negative, and it was clear that a severe streptococcal toxæmia was present.

On August 8th the patient was in a lethargic stuporose state, with mild pyrexia, and the condition was very grave. Several unhealthy crowned teeth were still present, and it was decided to remove one of these each day. The removal of the remaining infected teeth was followed by disappearance of the toxæmic condition, and the patient made a complete recovery. Dr. Geoffrey Evans made a most careful study of this case, and we were both of opinion that the removal of the remaining infected teeth, even though the condition was most grave, held out the only hope of recovery.

### LOCAL INFECTIVE CONDITIONS RESULTING FROM DENTAL INFECTIONS

Such conditions as stomatitis, tonsillitis, naso-pharyngeal infections, infections of the maxillary antra, cervical adentis, and Ludwig's angina have been observed.

### BLOOD CONDITIONS

A secondary anæmia, mild or severe in type is a common result of dental sepsis. The more severe types of anæmia are associated with streptococci of hæmolytic type. In many cases of pernicious anæmia a severe dental infection with hæmolytic streptococci is present. Hunter does not regard dental sepsis alone as a sufficient cause of pernicious anæmia, but rather as a predisposing cause to which an additional factor is added.

Leucocytosis is commonly present, and usually the differential count approximates to the normal. It may show variations in different cases, and in the same case at different stages of the disease.

Some acute cases show a marked relative lymphocytosis. This may be a true lymphocytosis, or simply an apparent lymphocytosis, due to a polymorphonuclear leucopenia.

Thus, in one acute case the blood examination on July 22, 1922, showed a leucocytosis of 16,600, with 17.5 per cent. of polymorphonuclears and 79.5 per cent. of lymphocytes. On August 3d the polymorphonuclears were 30 per cent. and the lymphocytes 65.7 per cent. On August 9th the polymorphonuclears were 64.29 per cent. and the lymphocytes 34.28 per cent.

Another similar acute case showed on February 9, 1921, a normal differential count, and on April 20th the lymphocytes were 52 per cent. and the polymorphonuclears 44 per cent.

An increase of the eosinophilia is uncommon. Two cases, however, have come to my notice.

The first patient (Case I) gave, on August 24, 1919, 20 per cent. of eosinophile leucocytes; on September 3d, 12 per cent.; on November 22d 15.05 per cent., and on January 9, 1920, 8.17 per cent.

Another patient, who had never resided in the tropics, showed 9 per cent. of eosinophile leucocytes in the differential count.

The presence of leucopenia in some cases has already been alluded to.

### CARDIO-VASCULAR COMPLICATIONS

Streptococcal infections of dental origin may cause phlebisis and venous thrombosis, and also arterio-sclerosis, which is not necessarily associated with an increase in the blood pressure. The changes in the arterial wall may give rise to narrowing of the lumen with symptoms of intermittent claudication, or even artero-thrombosis.

*Cardiac Conditions.* Tachycardia of toxic origin is often to be observed, and pericarditis, myocarditis, and myocardial degeneration may occur. Endocarditis when present may be of the simple type, such as occurs in acute rheumatism, but not infrequently dental sepsis gives rise to ulcerative endocarditis. Five such cases have been under my care during the past three years, where the origin of the infection appeared to be definitely the teeth.

### RESPIRATORY COMPLICATIONS

The streptococcal infection may give rise to laryngitis, tracheitis, and bronchitis. Pleurisy and empyema were described by Hunter, in 1900, as possible complications. Septic bronchopneumonia is a serious and not uncommon complication, and it may be followed by bronchiectasis, or lung abscess.

At the present time a patient is under my care in St. Mary's Hospital suffering from abscess of the lung, the result of very severe dental sepsis. Attention has been called to the adverse influence of dental infections in cases of pulmonary tuberculosis by Dr. R. C. Wingfield. He considers the removal of dental sepsis an essential preliminary in the treatment of pulmonary tuberculosis, and says that in such cases untreated oral sepsis may frequently turn the balance against the patient.

### GASTRO-INTESTINAL COMPLICATIONS

Dental sepsis is one of the commonest causes of gastric and intestinal dyspepsia. Hunter in 1899 laid stress on the frequency of toxic or infective gastritis, and he then expressed the opinion that dental sepsis was the probable cause in some cases of that rare condition "phlegmonous gastritis."

Gastric and duodenal ulcer probably result from septic infection, and the trend of opinion at the present time is that dental sepsis is a most important cause. In my experience it is rare to find a case of gastric or duodenal ulcer in which an adequate explanation of the cause can not be found in the condition of the teeth and gums, and I should say that dental sepsis was much the most important ætiological factor. The work of Rosenow in America gives strong support to the view that gastric and duodenal ulcer arise from streptococcal dental infection, and he has produced gastric and duodenal ulcer in animals from inoculation with cultures of human dental streptococci.

Appendicitis is, in many instances, due to a streptococcal infection, and several cases have recently been under my care in which the primary infection appeared undoubtedly to arise from the teeth.

Enteritis, with symptoms exactly like para-typhoid fever, including enlargement of the spleen and similar pyrexia, may undoubtedly result from streptococcal dental infections. The differential diagnosis can only be made by complete bacteriological investigations, and by the recognition of the dental disease for which often radiological examination is necessary. Three cases of this kind have been under my care.

Colitis, simple and ulcerative, is frequently due to a streptococcal infection, and in many of these cases the primary focus is undoubtedly connected with the teeth. Peri-apical dental necroses will often be found in intractable cases of colitis. Dr. N. Mutch has called attention to the frequency (84 per cent.) of pathogenic streptococci in the colon in an analysis of 200 cases of arthritis. In 52 per cent. of these cases he concluded that the primary focus was a dental infection.

### RENAL COMPLICATIONS

Nephritis has been described by many writers as sometimes resulting from dental sepsis.

### LIVER COMPLICATIONS

Toxic conditions of the liver are well known to result from streptococcal infections, and frequently slight jaundice and evidences of hepatic disturbance are observed in cases of dental infection. There can be no doubt that hepatic efficiency is often impaired as the result of the toxæmia from dental sepsis.

### SKIN COMPLICATIONS

Rashes of an erythematous, urticarial, papular, and eczematous type have been observed. Purpuric rashes may occur, especially where the streptococci are of the hæmolytic type.

### EYE COMPLICATIONS

Conjunctivitis, iritis, irido-cyclitis, episcleritis, retrobulbar neuritis, have all been described as resulting from dental infections. Undoubtedly vascular lesions, such as thrombosis of the central artery or vein of the retina, may be so caused. Of special interest is retinitis. Dr. Batty Shaw has recently called attention to the great importance of the toxic factor in this condition, and undoubtedly dental sepsis is not infrequently the cause.

### NERVOUS DISEASES

The toxæmia from dental infections may give rise to cerebral conditions such as abnormal mental states, melancholia, etc., and it is possible that inflammatory conditions, such as meningitis, may be so caused.

Spinal cord diseases, such as combined sclerosis with its associated anæmia, disseminated sclerosis, etc., are often due to toxic causes, and dental sepsis must be included amongst these.

Peripheral neuritis may occur from the streptococcal toxæmia of dental origin, and the sensory symptoms, tingling, and numbness of the hands and feet are of common occurrence. Local neuritis, such as sciatica, brachial neuritis, etc., is a common result of dental sepsis, but these are better included under the fibrositis group, because the cause is rather a perineuritis than a primary involvement of the nerve fibers.

### RHEUMATIC CONDITIONS

In a paper published in the *British Medical Journal* (June 4, 1921), I called attention to the great importance of infection of the teeth and gums in the causation of rheumatic conditions such as fibrositis and infective arthritis.

*Fibrositis.* The streptococcal infections so arising may give rise to the various forms of fibrositis—namely, panniculitis, inflammatory conditions of fasciæ, and aponeuroses—as occurs in lumbago and myalgic conditions; inflammations of tendons and ligaments, such as stiff neck, tender heels due to involvement of the plantar ligaments, Dupuytren's contractions of the palmar fascia; inflammations of tendon sheaths, arterio-synovitis; bursitis; Heberden's nodes; finger pads; fibrous nodules in subcutaneous tissue; local perineuritis and neuritis, as in sciatica and brachial neuritis. Fibrositis in some of its forms is the commonest occurrence in cases of dental and gum infections.

Non-specific infective arthritis, which includes the forms known as rheumatoid arthritis, arthritis deformans, osteo-arthritis, and chronic villous arthritis, is generally due to a streptococcal infection. Dr. Beddard, in a paper before this Society in October, 1918, expressed the opinion that 90 per cent. of these cases were due to infection arising from the teeth, and my personal experience agrees with this view.

It is well known that in cases of non-specific infective arthritis numerous bacteriological examinations have shown that no living organisms are to be found in the joints. It is probable that the streptococcal toxins give rise to the inflammatory conditions. The recent work of Dr. W. E. Gye, and Dr. E. H. Kettle has shown that marked proliferative changes can occur in organs such as the spleen, through the action of colloidal silica, without the presence of living micro-organisms.

Acute rheumatism is not commonly caused by dental infections, but several cases, have been described. The organism found by Poynton and Paine in numerous cases of acute rheumatism closely resembles the streptococcus salivarius and streptococcus fæcalis found in dental infections.

### GOUT

In some cases of gout dental sepsis is important, and should be removed, as far as possible, in every gouty patient. Dr. Llewellyn has expressed the opinion that gout is the result of a toxic idiopathy to certain toxic protein substances. Undoubtedly the streptococcal toxins of dental origin are in not a few cases the important causative factor.

### DIABETES

Streptococcal and other toxins may cause a toxic glycosuria, probably by impairment of the endocrine function of the pancreas. Dental sepsis is undoubtedly a factor in the causation of glycosuria in some cases, and it should always be removed in cases of diabetes. A rise in the carbohydrate tolerance has often been observed by me after the removal of dental sepsis in early cases of diabetes.

### SCURVY

This disease, due to vitamin deficiency, shows marked dental sepsis on its earliest appearance. During the war over 20,000 cases of scurvy occurred amongst Indian troops in Mesopotamia, many of which I personally examined, and in almost all very marked infection of the teeth and gums was present. The result of careful observation showed that if pre-existing dental sepsis was present in a marked degree, then such patients were very predisposed to develop scurvy.

### TREATMENT

The treatment of infections of the teeth and gums, and the diseases arising therefrom, does not come within the scope of this discussion. The most important general principles in the treatment of such infections have, however, been indicated, namely:

First, remove the focus of infection, either by extraction of teeth, or suitable treatment.

Secondly, it must be remembered that intestinal infections very frequently result from dental infection, and these may require treatment by such measures as Plombières colon irrigations, or an autogenous vaccine prepared from the streptococci found in the teeth and intestine.

PROPHYLAXIS

The early recognition of dental sepsis and its appropriate treatment would be one of the most important factors in greatly improving the health of the nation.—*British Medical Journal.*

